# Effect of n-Butyl Cyanoacrylate Coating on the Microhardness of MM-MTA After Exposure to Moisture and Human Blood

**DOI:** 10.3390/jfb17080410

**Published:** 2026-08-17

**Authors:** Jurica Matijević, Anja Baraba, Adna Vlahovljak Ferušić, Matea Vidov, Zoran Karlović, Ana Ivanišević

**Affiliations:** 1 University of Zagreb School of Dental Medicine, Department of Endodontics and Restorative Dentistry, Gundulićeva 5, 10000 Zagreb, Croatia; matijevic@sfzg.unizg.hr (J.M.); karlovic@sfzg.unizg.hr (Z.K.); 2 Private Dental Office Adenta, Bulevar Meše Selimovića 2C/3, 71000 Sarajevo, Bosnia and Herzegovina; adna.vlahovljak98@gmail.com; 3 Private Dental Office, Vivantadental, Avenida del Doctor Peset Aleixandre 142, 46002 Valencia, Spain; matea.vidov@gmail.com

**Keywords:** n-butyl cyanoacrylate, mineral trioxide aggregate, blood contamination, microhardness

## Abstract

The aim of this study was to evaluate the influence of n-butyl cyanoacrylate (NBCA) coating of freshly mixed mineral trioxide aggregate (MTA) on its microhardness after setting under conditions of moisture and human blood exposure. MicroMega MM-MTA samples were prepared using Teflon molds (6 mm × 4 mm). Four experimental groups (n = 4) were established: control (MTA + phosphate-buffered saline (PBS)), MTA coated with NBCA (PeriAcryl) + PBS, MTA + blood + PBS, and MTA coated with NBCA + blood + PBS. Samples assigned to the blood-exposure groups were exposed to human blood for 15 min, rinsed, and subsequently stored in PBS. Prior to microhardness testing, all samples were incubated in PBS for seven days. Microhardness was measured using a Vickers microhardness tester, with five indentations performed on each specimen (20 measurements per group). Data were analyzed using the Shapiro–Wilk test for normality, Levene’s test, and two-way ANOVA, followed by Tukey’s post hoc test. The analysis revealed significant differences among the experimental groups (*p* < 0.001). Post hoc analysis demonstrated statistically significant differences between tissue glue-coated and uncoated samples (*p* < 0.05). Within the limitations of this in vitro study, coating MTA with NBCA tissue glue during the setting period significantly increased its microhardness following exposure to human blood and PBS.

## 1. Introduction

Mineral trioxide aggregate (MTA) was introduced into dentistry in 1993 [1]. Although MTA was initially developed as a root repair material, its indications gradually expanded to a variety of clinical applications due to its excellent biocompatibility, sealing ability, and favorable clinical outcomes [2,3]. Today, MTA-based materials are used for retrograde root-end filling, repair of root resorption and root perforations, apexification, apical plug formation, direct pulp capping, restorative procedures, and root canal obturation [2]. Since its introduction, MTA has undergone numerous modifications aimed at improving its physical properties and handling characteristics while overcoming some of its shortcomings, including long setting time, tooth discoloration, and difficult handling [2,3,4,5,6,7]. One such modification is MicroMega MTA (MM-MTA). Compared with conventional MTA, MM-MTA has a considerably shorter setting time of approximately 20 min. Another advantage is its encapsulated delivery system, which provides a manufacturer-controlled powder-to-liquid ratio, ensuring optimal material properties and simplifying clinical application.

Under clinical conditions, MTA is frequently exposed to moisture and blood during or after placement. Previous studies have shown that blood contamination may adversely affect the setting reaction, mechanical properties, and adhesion of MTA to hard dental tissues [8,9,10]. Nekoofar et al. [8,9] demonstrated that exposure to blood significantly reduced both the compressive strength and microhardness of MTA. Furthermore, contamination of simulated furcation perforations with blood prior to MTA placement was shown to reduce the material’s resistance to displacement [10]. To simulate clinical conditions, phosphate-buffered saline (PBS) is commonly used as a storage medium for MTA in laboratory studies [11,12,13]. PBS is a synthetic body fluid whose inorganic composition resembles that of tissue fluids, although it lacks organic components. Storage of MTA in PBS has been shown to reduce coronal leakage [14], which may be attributed to the formation of a chemical bond between the apatite layer on the MTA’s surface and dentin, in the presence of phosphate-containing fluids [15,16]. Therefore, in vitro exposure of MTA to both PBS and human blood may provide a clinically relevant simulation of the conditions encountered during endodontic procedures.

Tissue glues are bioadhesive materials that adhere to biological tissues and prevent tissue separation by redistributing mechanical loads across the wound interface [17]. Among these materials, n-butyl cyanoacrylates (NBCAs) have been widely used for closure of skin, mucosal, and oral soft tissues [18]. Cyanoacrylates undergo rapid polymerization upon contact with water, tissue fluids, or blood, providing immediate adhesion and hemostasis while establishing a strong bond to both soft and hard tissues within approximately 60 s of application [19,20]. Furthermore, they are biodegradable, biocompatible, and exhibit antimicrobial properties [21]. Given these favorable characteristics, it may be hypothesized that application of a cyanoacrylate adhesive over freshly placed MTA could form a protective layer against contamination and washout, thereby potentially improving the material’s mechanical properties, including microhardness.

From a clinical perspective, the early setting phase of MTA is particularly important because the material is often placed in sites that are difficult to isolate completely from biological fluids. In contrast to conventional restorative procedures, where the operative field can usually be controlled more predictably, endodontic repair procedures frequently involve communication with periodontal or periapical tissues. In perforation repair, root-end filling, apexification, and vital pulp therapy, the material may be exposed to blood, tissue fluid, exudate, or irrigant residues shortly after placement. These conditions may compromise the surface characteristics, hydration behavior, and mechanical stability of calcium silicate-based materials before complete maturation has occurred [8,9,10,22,23]. Therefore, strategies that protect the material during the early phase of setting may be clinically valuable, particularly if they do not interfere with the bioactive interaction between the cement and the surrounding tissues.

Microhardness testing is commonly used as an indirect indicator of the degree of material maturation and surface integrity [24]. Although microhardness does not fully represent the complex mechanical behavior of hydraulic calcium silicate cements in vivo, it provides useful information regarding the resistance of the material surface to deformation. A reduction in microhardness may indicate incomplete hydration, increased porosity, or surface degradation, all of which could be relevant for clinical performance. In situations where the coronal or exposed surface of MTA is subjected to condensation pressure, restorative procedures, or early contamination, improved surface hardness may contribute to better material stability. For this reason, microhardness remains a relevant experimental outcome when investigating modifications intended to improve the early physical properties of MTA-based materials.

The concept of applying a protective external coating over freshly placed MTA is clinically attractive because it does not necessarily require modification of the cement itself. Altering the composition of calcium silicate materials may affect their hydration, ion release, bioactivity, or biocompatibility [25,26,27]. In contrast, a superficial coating may act as a temporary stabilizing layer while preserving the core biological properties of the underlying cement. NBCA tissue adhesive is especially interesting in this context because it polymerizes rapidly in the presence of moisture and blood, forms a thin adherent film, and has already been used in oral and surgical applications. If such a coating can protect the surface of freshly mixed MTA without impairing its clinical function, it may represent a simple adjunctive approach in challenging situations where bleeding or moisture control is difficult.

Despite the potential clinical advantages of NBCA, evidence regarding its interaction with calcium silicate-based endodontic materials remains limited. Camargo et al. evaluated the microhardness and sealing ability of materials used for root perforation repair and investigated the effect of cyanoacrylate application as a protective coating over MTA [28]. The authors reported that cyanoacrylate application did not significantly improve the microhardness or sealing ability of MTA compared with MTA alone [28]. However, their study evaluated conventional MTA in a perforation repair model, with samples kept in 100% humidity, and did not investigate the influence of cyanoacrylate coating on the behavior of freshly mixed calcium silicate cement under clinically relevant conditions involving blood exposure or exposure to body fluid [28].

In the present study, NBCA was applied as a protective coating over freshly mixed MM-MTA with the purpose of creating a barrier between the cement surface and the surrounding environment, particularly under conditions of potential blood contamination. Although blood contamination has been shown to influence the physical properties, microstructure, and surface microhardness of MTA and other hydraulic calcium silicate-based materials [24,29], it remains unclear whether the application of an NBCA coating can preserve or modify the mechanical maturation of MM-MTA when exposed to blood during setting. Therefore, an important knowledge gap remains, as the effect of NBCA application as a protective coating on the surface microhardness of MM-MTA under clinically relevant conditions, like moisture and human blood exposure, has not been established.

The aim of this study was to evaluate the influence of n-butyl cyanoacrylate tissue glue application on the microhardness of freshly mixed MM-MTA after setting under conditions of moisture and human blood exposure. The specific objectives were to compare the microhardness of MM-MTA set under moist conditions or following blood exposure, with and without NBCA tissue glue application, and to determine whether an NBCA coating exerts a protective effect on the underlying MM-MTA during the early setting period, thereby influencing the microhardness of the matured material.

## 2. Materials and Methods

The study was approved by the Ethics Committee of the School of Dental Medicine, University of Zagreb (251-60-4/119-20).

### 2.1. Materials Used in the Study

Materials used in the study were encapsulated MM-MTA (MicroMega/Coltene, Besançon, France) and PeriAcryl^®^ 90 HV Oral Tissue Adhesive (GluStitch Inc., Delta, BC, Canada) (Table 1). The storage medium was phosphate-buffered saline (PBS), pH 7.4 (Invitrogen/Thermo Fisher, Waltham, MA, USA).

### 2.2. Preparations of the Samples and Experimental Groups

**Figure 1:**
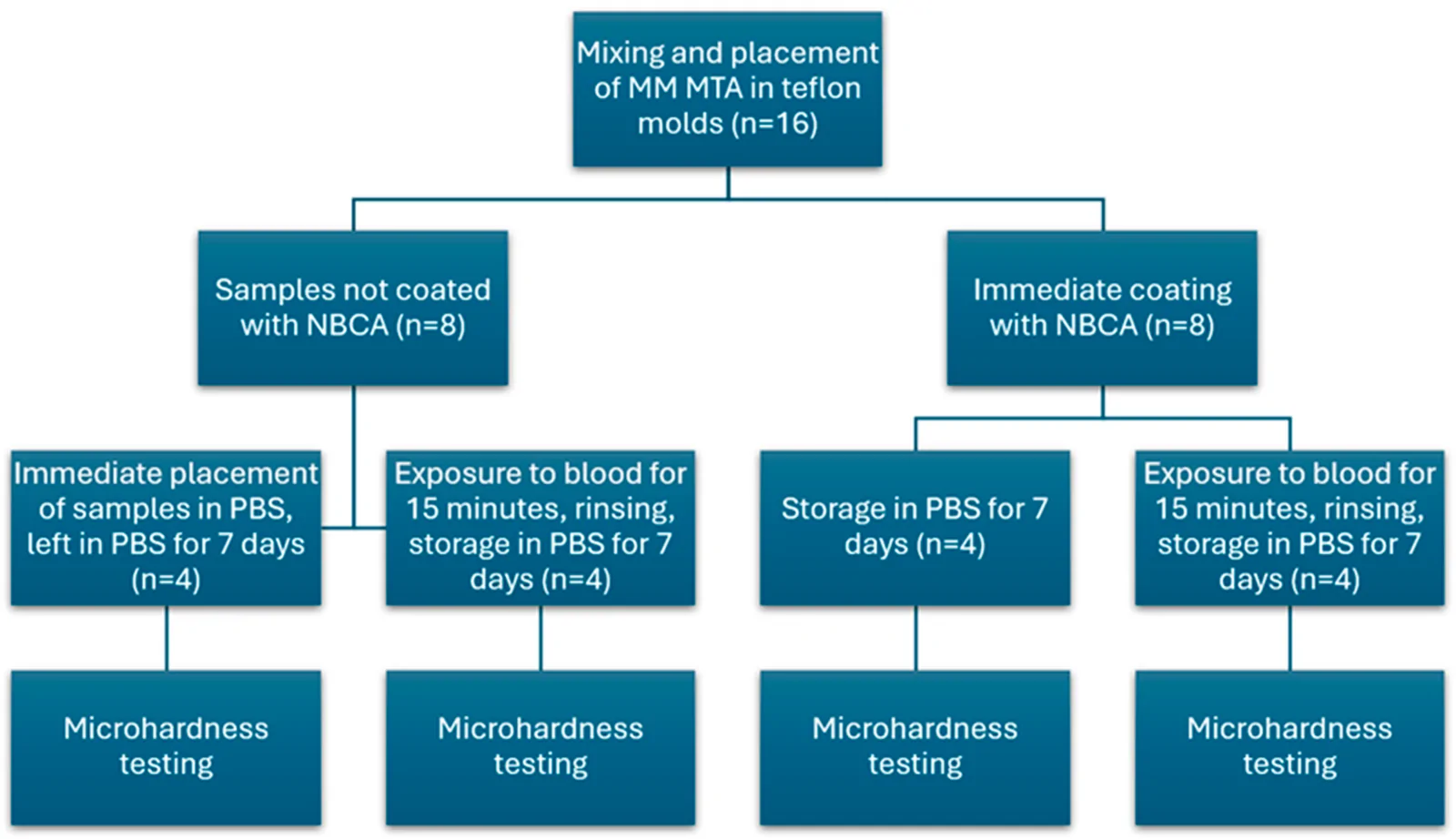
Methodological flow diagram for the study where Vickers microhardness was measured in four experimental groups: 1. MTA samples adapted in the molds and stored in PBS; 2. MTA samples exposed to fresh anticoagulated human blood for 15 min, rinsed with 5 mL of PBS, and stored in PBS; 3. MTA samples coated with PeriAcryl immediately after mixing and stored in PBS; and 4. MTA samples coated with PeriAcryl immediately after mixing, exposed to fresh human blood for 15 min, rinsed with 5 mL of PBS, and incubated in PBS at 37 °C for 7 days.

MM-MTA encapsulated material was mixed according to the manufacturer’s instructions and extruded into standardized teflon molds, 6 mm in diameter and 4 mm in height, placed on a glass base. The material was condensed using a round stainless steel condenser to ensure proper adaptation and a flat surface. Half of the samples were immersed in 20 mL of freshly drawn human blood for 15 min (Figure 1). Venipuncture was performed by trained personnel using a Vacuette blood collection kit (Greiner Bio-One, Kremsmünster, Austria), according to WHO guidelines [30]. Fresh peripheral blood obtained from a single healthy volunteer, who was one of the study investigators, was used for all blood-exposed specimens. The same donor was used throughout the experiment to standardize the contamination medium and minimize inter-donor biological variability. The donor was informed about the purpose and possible benefits of the study and filled out an informed consent form.

A total of four groups were prepared, each consisting of four samples (n = 4): 1. MTA samples adapted in the molds and stored in PBS (control group); 2. MTA samples adapted in the molds, coated with PeriAcryl immediately afterwards, and stored in PBS; 3. MTA samples adapted in the molds, exposed to fresh anticoagulated human blood for 15 min, rinsed with 5 mL of PBS, and stored in PBS; and 4. MTA samples adapted in the molds, coated with PeriAcryl immediately afterwards, exposed to fresh human blood for 15 min, rinsed with 5 mL of PBS, and incubated in PBS at 37 °C for 7 days (ES 120, Nüve, Ankara, Turkey) (Figure 1). MTA samples in all groups were left in the molds throughout the experiment. Measuring microhardness after 7 days enabled the evaluation of the effect of early blood/PBS exposure and NBCA coating on the microhardness of the material after a standardized maturation period.

### 2.3. Microhardness Testing

Before Vickers microhardness testing, the cured NBCA coating was mechanically removed by gentle scraping. As the NBCA layer was not adhesively bonded to the MTA surface, it could be completely detached in one piece using a Heidemann spatula, without polishing. The specimens were then visually inspected, and the indentations were performed directly on the exposed MTA surface rather than through the NBCA layer. Microhardness of the samples was measured using a Vickers microhardness tester (KB Prüftechnik GmbH, Hochdorf-Assenheim, Germany), applying a 1 kg load for 10 s. Five indentations were made on each sample, resulting in 20 measurements per experimental group. The values were recorded in Vickers hardness units (HV). The 4 mm specimen thickness was chosen to ensure uniform sample preparation, adequate material mass, and a flat testing surface while minimizing the possible influence of the underlying glass substrate during indentation. With a 4 mm thickness, it was ensured that the glass support beneath the MTA material would not influence the result.

### 2.4. Sample Size Calculation

Sample size estimation was performed using G*Power software, version 3.1.9.7, for a 2 × 2 factorial ANOVA design. Since only limited previous data were available regarding the effect of NBCA coating on the microhardness of freshly mixed MM-MTA exposed to PBS and human blood, the calculation was based on the detection of a large effect size for the main effect of NBCA coating. Using α = 0.05, statistical power of 0.80, and a large effect size, the minimum required sample size was lower than the final number of specimens included in the study.

As a sensitivity check based on the observed main effect of NBCA coating, the partial η^2^ value of 0.735 corresponded to Cohen’s f = 1.665. Under these assumptions, the minimum required total sample size was 7 specimens. To maintain a balanced experimental design and account for specimen-level variability, four specimens were included in each experimental group, resulting in a total of 16 specimens. Five indentations were performed on each specimen; however, the specimen was considered the experimental unit for the primary statistical analysis.

### 2.5. Statistical Analysis

Statistical analysis was performed using specimen-level mean Vickers microhardness values using SPSS 17.0, Excel 2016, and ChatGPT 5.4.

Since five indentations were performed on each specimen, the individual indentation measurements were not considered independent experimental units. Therefore, the mean microhardness value was calculated for each specimen and used for the primary statistical analysis. The normality of model residuals was assessed using the Shapiro–Wilk test, and homogeneity of variances was evaluated using Levene’s test. The experimental design was analyzed as a 2 × 2 factorial model, with NBCA coating and blood exposure as fixed factors. The main effects of NBCA coating and blood exposure, as well as their interaction, were evaluated using two-way analysis of variance (ANOVA). When appropriate, Tukey’s post hoc test was used for multiple comparisons among the four experimental groups. As a sensitivity analysis, a linear mixed-effects model was additionally fitted using individual indentation values, with NBCA coating, blood exposure, and their interaction included as fixed effects, and specimen included as a random intercept to account for repeated measurements within the same specimen. Data are presented as mean ± standard deviation. The level of statistical significance was set at α = 0.05.

## 3. Results

No significant deviations from normality (*p* > 0.05, Table 2) or homogeneity of variances (Levene’s test, *p* = 0.719) were detected for the specimen-level analysis.

Individual indentation values are presented descriptively in Table 3. For inferential statistics, specimen-level mean values and a mixed-effects model were used to avoid treating repeated indentations from the same specimen as independent experimental units.

Two-way ANOVA performed on specimen-level mean microhardness values showed a significant main effect of NBCA coating on Vickers microhardness (F = 33.28, *p* = 0.000089, partial η^2^ = 0.735). In contrast, blood exposure did not have a significant effect on microhardness (F = 0.14, *p* = 0.712866, partial η^2^ = 0.012), and the NBCA coating × blood exposure interaction was not statistically significant (F = 0.45, *p* = 0.516746, partial η^2^ = 0.036), but this result should be interpreted cautiously because the small number of specimens per group provided limited power to detect an interaction effect.

**Figure 2:**
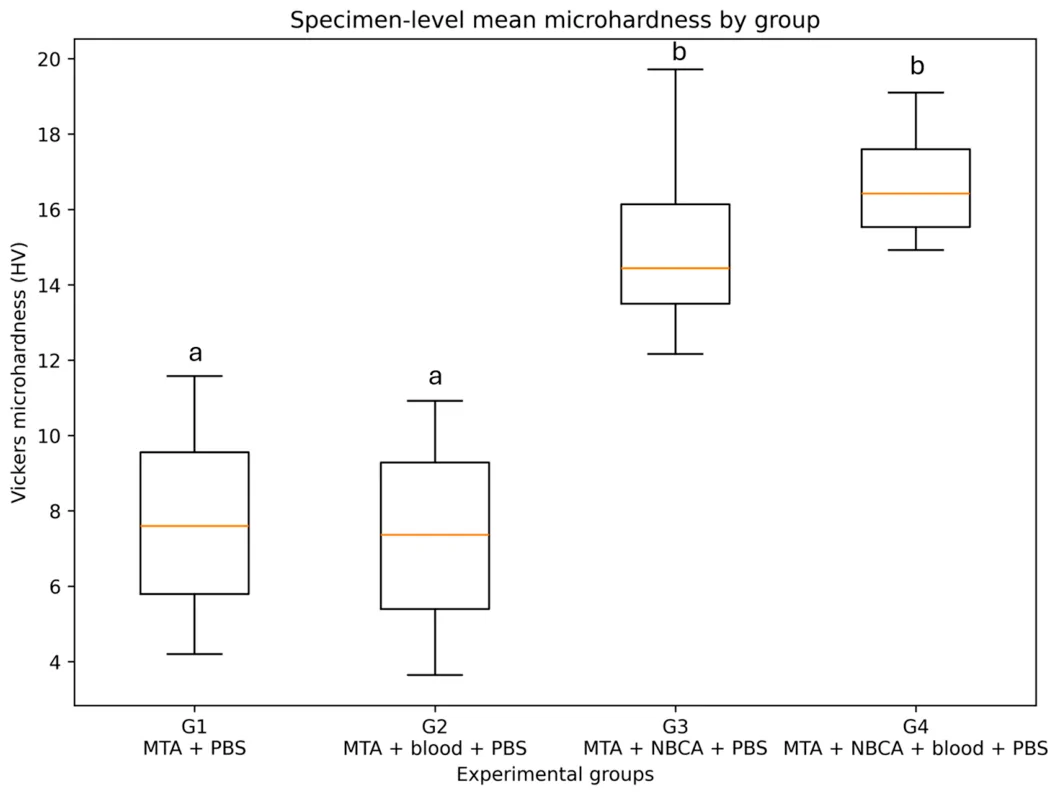
Specimen-level Vickers microhardness values of MM-MTA in the experimental groups. The horizontal line within each box represents the median, boxes represent the interquartile range, and whiskers indicate the minimum and maximum values. Different lowercase letters indicate statistically significant differences among groups according to Tukey’s post hoc test (*p* < 0.05). Groups sharing the same letter are not significantly different. MTA, mineral trioxide aggregate; NBCA, n-butyl cyanoacrylate; PBS, phosphate-buffered saline.

Tukey’s post hoc test showed that NBCA-coated groups had significantly higher microhardness values than the corresponding uncoated groups. The comparison between MTA + PBS and MTA + NBCA + PBS was significant (mean difference = 7.45 HV, *p* = 0.016408, Hedges’ g = 2.02), as was the comparison between MTA + PBS and MTA + NBCA + blood + PBS (mean difference = 8.97 HV, *p* = 0.004550, Hedges’ g = 3.00). Similarly, MTA + blood + PBS differed significantly from MTA + NBCA + PBS (mean difference = 7.87 HV, *p* = 0.011443, Hedges’ g = 2.14) and from MTA + NBCA + blood + PBS (mean difference = 9.40 HV, *p* = 0.003206, Hedges’ g = 3.15). No statistically significant difference was observed between the two uncoated groups, MTA + PBS and MTA + blood + PBS (mean difference = −0.43 HV, *p* = 0.996738, Hedges’ g = −0.12), or between the two NBCA-coated groups, MTA + NBCA + PBS and MTA + NBCA + blood + PBS (mean difference = 1.53 HV, *p* = 0.879589, Hedges’ g = 0.51) (Figure 2).

The linear mixed-effects model confirmed the findings of the primary specimen-level analysis. NBCA coating remained significantly associated with increased microhardness (*p* = 0.000310), whereas blood exposure (*p* = 0.836860) and the NBCA coating × blood exposure interaction (*p* = 0.504099) were not significant. The intraclass correlation coefficient was 0.345, indicating that approximately 34.5% of the total variance was attributable to between-specimen differences.

## 4. Discussion

The present study demonstrated that coating MTA with an n-butyl cyanoacrylate tissue adhesive significantly increased its microhardness. These findings may be clinically relevant because hydraulic calcium silicate cements are frequently used in procedures performed in the presence of moisture and bleeding, including furcation perforation repair, direct pulp capping, and root-end surgery. Under such conditions, maintaining the physical integrity of the material during its initial setting period is essential for predictable clinical outcomes. In the present study, blood exposure and NBCA application were performed during the early setting phase of MTA, whereas microhardness was measured after 7 days of maturation. Therefore, the findings represent the persistent consequences of events/interventions occurring during early setting rather than the immediate dynamics of material hardening. The 7-day endpoint was selected to determine whether early contamination or surface protection affected the properties of the subsequently matured material.

Most studies have focused on improving the properties of hydraulic calcium silicate cements themselves [31,32,33], whereas the concept of enhancing their performance through the application of an external adhesive retention layer has received little attention. According to the available literature, information regarding the application of n-butyl cyanoacrylate (NBCA) over hydraulic calcium silicate cement and its influence on their mechanical properties is scarce [28]. Previous studies have primarily focused on the sealing ability of cyanoacrylate when used as a root-end filling material rather than as a coating. In vitro investigations demonstrated that cyanoacrylate used for retrograde cavity filling exhibited lower microleakage than MTA, with acceptable biocompatibility [34,35]. However, to the best of our knowledge, no studies have evaluated the effect of an NBCA coating on the mechanical properties of hydraulic calcium silicate cements.

Hydraulic calcium silicate cements are considered materials of choice for regeneration-oriented endodontic procedures because of their favorable biological properties [36,37]. Nevertheless, limitations such as susceptibility to washout during setting, surface porosity, solubility, fluid uptake, marginal adaptation, and microleakage continue to be reported [5,6,38]. Owing to its film-forming properties, an NBCA coating may have the potential to influence some of these characteristics by protecting the material surface during the early stages of setting. Such a coating could theoretically improve surface integrity and resistance to disintegration in moist environments. Therefore, evaluating the effect of NBCA application on the physical and mechanical properties of hydraulic calcium silicate cements, including microhardness, is an important first step in assessing the feasibility of this approach.

PeriAcryl, which is chemically based on n-butyl cyanoacrylate (NBCA), was developed primarily as a topical oral tissue adhesive. Owing to its relatively slow degradation compared with short-chain cyanoacrylates, NBCA has demonstrated acceptable biocompatibility in a variety of dental and medical applications [19,20,39]. It has been successfully used in oral surgery and periodontology [17] and has even been investigated as a pulp-capping material because of its sealing ability and capacity to support dentin bridge formation [40,41].

Despite the favorable biocompatibility of NBCA, hydraulic calcium silicate cements remain biologically superior for regenerative endodontic procedures. In addition to exhibiting low cytotoxicity, these materials are bioactive and capable of promoting tissue repair and regeneration through calcium ion release, mineralization, osteogenic and cementogenic differentiation, and hard-tissue formation [3,36,37]. Because these biological effects depend largely on direct interaction between the material and surrounding tissues [7], the clinical value of an NBCA coating is likely to differ according to the intended application. In root-end surgery, where tissue contact with the root-end filling material is critical, enhanced microhardness alone may not necessarily translate into improved biological outcomes. In contrast, in procedures such as root perforation repair and direct pulp capping, where NBCA may function primarily as an external stabilizing layer while the hydraulic calcium silicate cement remains in direct contact with the tissues, the improved mechanical properties observed in the present study may be of greater clinical relevance.

The findings of the present study may be particularly relevant for root perforation repair and direct pulp capping procedures. Before placing MTA, hemostasis should ideally be achieved, which in pulp capping typically takes up to 5 min using appropriate hemostatic measures [42]. The time required to achieve hemostasis in root perforations depends on the size and location of the perforation and the degree of periodontal inflammation, and no standardized duration has been established in the literature. In the present study, in which MM-MTA was adapted and condensed in molds for several minutes (coated with NBCA) and then exposed to blood for 15 min before rinsing and storage, it represents a clinically plausible scenario with prolonged bleeding. It simulates a situation in which complete hemostasis is difficult to achieve, or blood re-enters the perforation site during the early setting phase of the cement. Since the first 15–20 min correspond closely to the period of initial hydration and clinical setting [43], this exposure is highly relevant when evaluating the effects of blood contamination on the material’s final microhardness. Application of NBCA to the coronal or external surface of the material may provide additional stabilization, particularly in large perforations or challenging clinical situations where complete hemostasis and moisture control are difficult to achieve. In such cases, the hydraulic calcium silicate cement would remain in direct contact with the tissues and retain its bioactive function, whereas NBCA would serve primarily as a protective retention layer. The improved microhardness observed in the present study may therefore indicate enhanced resistance to surface deterioration and material displacement during the initial setting. Furthermore, external NBCA coating could potentially protect the underlying hydraulic calcium silicate cement during placement of the definitive restoration and reduce the risk of disruption of the material-restoration interface. Recent evidence suggests that the choice of restorative material placed over MTA may influence interfacial integrity, particularly because hydraulic calcium silicate cements develop a highly alkaline environment, whereas some restorative materials, including conventional glass ionomer cements and certain adhesive systems, exhibit an acidic setting reaction [44]. Although the influence of NBCA on this interface has not been investigated, its rapid polymerization and chemically neutral nature may make it an interesting candidate for further study as an intermediate protective layer.

Several limitations of the present study should be acknowledged. The sample size was limited, and although multiple indentations were performed on each specimen, the true experimental unit was the specimen itself. This limitation was addressed statistically by using specimen-level mean values for the primary analysis and by applying a mixed-effects model as a sensitivity analysis. Nevertheless, the observed absence of statistical significance of blood × NBCA interaction does not establish that the effect of NBCA was independent of blood exposure, because the small number of specimens per group provided limited power to detect an interaction effect. Studies with larger, prospectively determined sample sizes are needed to confirm the magnitude and consistency of the observed effect. Another limitation may be the use of blood from a single donor, which standardizes the contamination medium but limits the generalizability of the findings. Donor-specific differences in hematocrit, coagulation factors, plasma proteins, and other blood parameters may affect the interaction between blood and the tested materials. Furthermore, the donor was one of the study investigators, which was transparently disclosed but may be perceived as a potential source of bias. Future studies should include blood from multiple independent donors and should be specifically designed and powered to assess inter-donor variability. Furthermore, only the 7-day endpoint microhardness was evaluated. Although this design enabled assessment of the lasting effects of conditions/interventions during the early setting phase (blood/PBS exposure and NBCA coating), it did not characterize the setting dynamics under conditions of blood and moisture. Additional measurements at 30 min, 1 h, 24 h, and later maturation periods would provide a more comprehensive understanding of the setting process and the time-dependent protective effect of NBCA.

The present study was designed as an initial proof-of-concept investigation and was limited to the evaluation of Vickers microhardness. Although surface microhardness is a relevant parameter for assessing the mechanical condition of the material surface after setting, it does not provide a complete characterization of the material’s performance. Further studies are required to determine whether NBCA coating also affects other clinically relevant properties, including compressive strength, sealing ability, washout resistance, solubility, degradation in simulated body fluid, interaction with final restorative materials, ion release, and biological response.

## 5. Conclusions

The present study introduces a novel concept in which a bioactive hydraulic calcium silicate cement is combined with an external NBCA retention layer. Within the limitations of this preliminary in vitro study, NBCA coating significantly increased the Vickers microhardness of MM-MTA under the tested experimental conditions and after seven days of storage in PBS. Since only surface microhardness was evaluated, no conclusions can be drawn regarding the overall mechanical performance and biological response. Further studies are required to determine whether the observed increase in microhardness of MTA coated with NBCA translates into clinical effectiveness of this approach.

## Figures and Tables

**Table 1:** Type of material, manufacturer, and composition of the materials used in the study.

	Type of the Material	Manufacturer	Composition
MM MTA LOT 72201046	Calcium-silicate cement	Micro-Mega (Besançon, France)	Powder: Tricalcium silicate, dicalcium silicate, tricalcium aluminate, calcium carbonate, calcium sulfate, and bismuth oxide. Liquid: Distilled water.
PeriAcryl^®^ 90 HV Oral Tissue Adhesive LOT GBOP23124-1024	Cyanoacrylate oral tissue adhesive	GluStitch Inc., Delta, BC, Canada	Active Monomer Ingredients: n-Butyl Cyanoacrylate, 2-Octyl Cyanoacrylate Additives and Modifiers: Thickening/viscosity Agents, violet colorant

**Table 2:** Results of the Shapiro–Wilk normality test.

Group	Shapiro–Wilk W	*p*
MTA + PBS	0.988	0.947
MTA + blood + PBS	0.984	0.927
MTA + NBCA + PBS	0.918	0.525
MTA + NBCA + blood + PBS	0.959	0.776

MTA, mineral trioxide aggregate; NBCA, n-butyl cyanoacrylate; PBS, phosphate-buffered saline.

**Table 3:** Descriptive statistics based on individual indentation measurements. Five measurements were done on each sample, resulting in 20 measurements in each group. The table summarizes all individual Vickers indentation values within each experimental group.

Group	n	Mean ± SD (HV)	Median (Q1–Q3)	Min-Max	95% CI
MTA + PBS	20	7.75 ± 3.09	7.50 (4.80–9.95)	3.80–14.10	6.30–9.19
MTA + blood + PBS	20	7.32 ± 3.00	6.60 (5.08–9.95)	2.90–11.80	5.92–8.72
MTA + NBCA + PBS	20	15.19 ± 3.55	13.70 (12.85–17.05)	10.20–23.30	13.53–16.85
MTA + NBCA + blood + PBS	20	16.71 ± 5.94	16.00 (13.12–17.50)	9.10–35.30	13.93–19.50

HV, Vickers hardness; NBCA, n-butyl cyanoacrylate; PBS, phosphate-buffered saline; SD, standard deviation; CI, confidence interval.

## Data Availability

The original contributions presented in this study are included in the article. Further inquiries can be directed to the corresponding authors.
